# Nanoporous ZIF-8 Microparticles as Acetylcholinesterase and Alkaline Phosphatase Mimics for the Selective and Sensitive Detection of Ascorbic Acid Oxidase and Copper Ions

**DOI:** 10.3390/bios12111049

**Published:** 2022-11-21

**Authors:** Guo-Ying Chen, Shi-Jun Yin, Li Chen, Xi Zhou, Feng-Qing Yang

**Affiliations:** 1 Department of Pharmaceutical Engineering, School of Chemistry and Chemical Engineering, Chongqing University, Chongqing 401331, China; 2 Key Laboratory of Optoelectronic Technology and Systems, Ministry of Education, Key Disciplines Lab of Novel Micro-Nano Devices and System Technology, College of Optoelectronic Engineering, Chongqing University, Chongqing 400044, China

**Keywords:** alkaline phosphatase, acetylcholinesterase, ascorbic acid oxidase, copper ion, zeolitic imidazolate framework-8, colorimetric detection

## Abstract

In this study, the alkaline phosphatase (ALP)-like activity of zeolitic-imidazolate framework-8 (ZIF-8) is reported for the first time. Then, colorimetric sensors for the ascorbic acid oxidase (AAO) and copper ion (Cu^2+^) detection were developed based on the acetylcholinesterase (AChE)- and ALP-like activities of ZIF-8. The ZIF-8 has good mimetic enzyme activity and exhibits high affinity to the substrates. Its AChE- and ALP-like activities also have good reusability and storage stability. Good linear dependences are obtained in the range of 1.3−250.0 μM (AChE-like activity-based) and 4.5−454.5 μM (ALP-like activity based) for Cu^2+^ detection. Furthermore, good linear dependence is also obtained based on the ALP-like activity of ZIF-8 for AAO detection in the range of 2.3−454.5 U/L. Their limits of detection (LODs) are calculated to be 0.7 µM, 2.8 µM, and 1.8 U/L, respectively. Finally, the sample spiked recoveries of Cu^2+^ in tap water, Cu^2+^, and AAO in human serum and rabbit plasma were measured, and the results are in the range of 80.0−119.3%. In short, the preparation of ZIF-8 is simple, environmentally friendly, and harmless, and can realize highly selective detection of AAO and Cu^2+^ in an efficient and fast process.

## 1. Introduction

Zeolitic-imidazolate framework-8 (ZIF-8), which is constructed by tetrahedral coordination of zinc cations (Zn^2+^) to the nitrogen in 2-methylimidazole linkers, is one of the representative members of metal-organic frameworks (MOFs). ZIF-8 has a uniform porous structure, high surface area and porosity, and good chemical stability [1]. Compared with other MOFs, the preparation of ZIF-8 has the characteristics of being a rapid, simple, and green synthesis process that utilizes a harmless solvent under mild conditions [2,3]. ZIF-8 has been extensively used in the fields of gas storage and capture, membrane technology, energy storage, and analytical chemistry [4]. Interestingly, ZIF-8 possesses various enzyme-like activities, such as human carbonic anhydrase-, promiscuous esterase-, and acetylcholinesterase (AChE)-like activities [5]. Although, to date, it has rarely been applied in the field of mimetic enzymes, ZIF-8 has great potential due to its unique and stable properties.

Cu^2+^, which is the most important transition metal ion found in both humans and animals, plays important roles in various physiological processes and acts as a catalytic cofactor for various enzymes, such as tyrosinase and superoxide dismutase [6]. However, the abnormal intake of Cu^2+^ can result in several neurodegenerative diseases, such as Alzheimer’s disease, Menke’s disease, and Wilson’s disease [7]. Therefore, the development of a highly sensitive method for the determination of Cu^2+^ is considerably important.

Ascorbate oxidase (AAO) is an oxidoreductase that can catalyze ascorbic acid (AA) to generate dehydroascorbate (DHA) and water in the presence of oxygen. AAO is widely distributed in the cytoplasm or bound to the cell wall in plants in the form of membrane binding that can regulate the redox state and growth of plant cells [8]. Although many methods and sensing strategies have been reported for the detection of AA, there are few reports related to the detection of AAO activity [9]. Therefore, the construction of a simple and sensitive method for AAO activity evaluation is significant.

**Figure 1:**
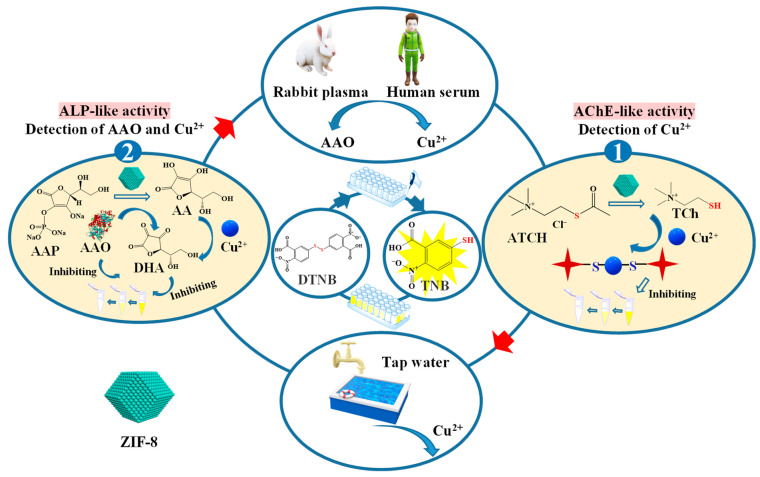
Schematic illustration for the detection of AAO and Cu^2+^ based on the AChE- and ALP-like activities of ZIF-8.

In this study, based on our preliminary investigations, a simple and reliable colorimetric strategy was designed and optimized for Cu^2+^ and AAO detection based on the AChE- and ALP-like activities of ZIF-8. As shown in Figure 1, the AChE- and ALP-like activities of ZIF-8 are used to detect Cu^2+^ and AAO in biological and tap water samples. The AChE-like activity of ZIF-8 can hydrolyze acetylthiocholine chloride (ATCh) to thiocholine (TCh), which will further react with 5,5′-dithiobis-(2-nitrobenzoic acid) (DTNB) to produce 2-nitro-5-thiobenzoic acid (TNB), which has a maximum absorption peak at 405 nm. When adding Cu^2+^ to the system, Cu^2+^ can interact with TCh to form a complex, and less TCh can react with DTNB to produce TNB. Conversely, the ALP-like activity of ZIF-8 can hydrolyze L-ascorbic acid 2-phosphate trisodium salt (AAP) to AA, which will further react with DTNB to generate TNB. When adding Cu^2+^ to the reaction system, the AA will react with Cu^2+^ to form Cu^+^. Thus, the addition of Cu^2+^ can inhibit this process by reducing the reaction of AA with DTNB. In addition, less AA can act with DTNB to generate TNB when AAO is presented, which can catalyze AA to DHA. Therefore, the determination of Cu^2+^ and AAO can be realized based on the enzyme-like activities of ZIF-8. Under the optimized conditions, the linear relationship between the Cu^2+^ and AAO concentrations and the inhibition rate of ZIF-8 activity was determined. Then, the developed method was applied to detect Cu^2+^ and AAO in tap water, human serum, and rabbit plasma samples. This study provides a new method for multiple analytes analysis, including metal ions and enzyme activity assay.

## 2. Materials and Methods

### 2.1. Materials and Reagents

Magnesium sulfate heptahydrate (MgSO_4_·7H_2_O), sodium chloride (NaCl), and potassium chloride (KCl) were obtained from Chengdu Chron Chemicals Co., Ltd. (Chengdu, China). Copper (II) sulfate pentahydrate (CuSO_4_·5H_2_O) was purchased from Chongqing Chuandong Chemical Group Co., Ltd. (Chongqing, China). The Calcium chloride (CaCl_2_) was the product of Tianjin Damao Chemical Reagent Factory (Tianjin, China). Zinc nitrate hexahydrate (Zn (NO_3_)_2_·6H_2_O, 99%) was purchased from Adamas-beta Co., Ltd. (Shanghai, China). Melamine, DTNB, and zinc acetate dihydrate (C_4_H_6_O_4_Zn∙2H_2_O) were purchased from Macklin (Shanghai, China). The rabbit plasma (with sodium citrate as the anticoagulant), D (+)-glucose, D (+)-lactose, 2-methylimidazole, vitamin B6 (VB6), vitamin B3 (VB3), vitamin B5 (VB5), ATCh (≥99), and AAO (263 U/mg) were purchased from Shanghai YuanYe Biological Technology Co., Ltd. (Shanghai, China). L (+)-Glutamic acid and AAP were purchased from Shanghai Aladdin Biochemical Technology Co., Ltd. (Shanghai, China). L-Lysine was purchased from Chengdu Huaxia Chemical Reagent Co., Ltd. (Chengdu, China). D-phenylalanine was purchased from TargetMol (Boston, MA, USA). Normal human serum was purchased from Beijing Solarbio Science & Technology Co., Ltd. (Beijing, China).

### 2.2. Instruments

UV-Vis analysis was carried out on a UV-5500 PC spectrophotometer (Shanghai Metash Instruments Co., Ltd., Shanghai, China) (wavelengths ranging from 400 nm to 500 nm were used in this study). The tabletop low-speed centrifuge L420 used in this study was obtained from Hunan Xiang Yi Laboratory Instrument Development Co., Ltd. (Changsha, China) (the centrifugal force of 2259× *g* is used in this study). A drying oven (DHG-9146A, Longyue Instrument Equipment, Shanghai, China) was used in the temperature-controlling process. The vacuum drying oven used in the preparation of ZIF-8 was obtained from Yiheng Scientific Instruments Co., Ltd., Shanghai, China (the temperature is controlled at 50 °C). The structure of ZIF-8 was characterized using a field-emission scanning electron microscopy (SEM) (JSM-7600F, JEOL Ltd., Tokyo, Japan) (the magnifications are ×10,000 and ×20,000). A nitrogen sorption study was performed on a Quadrasorb 2 MP (Kantar, New York, NY, USA) specific surface and aperture analyzer (with a degassing temperature of 150 °C). The Fourier-transform infrared (FT-IR) spectrum of ZIF-8 was recorded using a Nicolet iS50 (Thermo Scientific Inc., Waltham, MA, USA). The sample’s X-ray diffraction (XRD) patterns were acquired using an X’ pert Powder diffractometer (Malvern Panalytical Ltd., Eindhoven, The Netherlands) with secondary beam graphite monochromated Cu Kα radiation. In addition, the ultrapure water used throughout this study was purified by a water purification system (ATSelem 1820A, Antesheng Environmental Protection Equipment, Chongqing, China).

### 2.3. Synthesis of ZIF-8

The synthesis of ZIF-8 was carried out in line with the previously reported method [1]. Firstly, 4 mL of C_4_H_6_O_4_Zn∙2H_2_O (0.4 M) and 40 mL of 2-methylimidazole (0.8 M) were added to a 100 mL beaker, and then placed in an oven and reacted at 30 °C for 2 h. The prepared material was purified through centrifugation for 5 min at 2259× *g* and rinsed with ultra-pure water twice. Finally, the obtained ZIF-8 was placed in a vacuum oven and kept at 50 °C overnight.

### 2.4. Enzyme-like Activity of ZIF-8

The AChE-like activity of ZIF-8 was analyzed through the cascade reactions of ATCh and DTNB. Firstly, ZIF-8 can catalyze the ATCh to generate TCh, which will further react with DTNB to generate TNB, which has a 405 nm maximum absorption peak. A 4 mg sample of ZIF-8, 50 μL of ultra-pure water, 150 μL of 11.3 mM of ATCh (prepared in 10.0 mM of phosphate buffer, pH 7.5), and 400 μL of 13.3 mM of DTNB (prepared in ethanol) were mixed in a 1.5 mL centrifuge tube, which was then incubated at 60 °C for 15 min. The absorbance (405 nm) of the supernatant was recorded after the mixture had been centrifuged on a handheld mini centrifuge for 1 min. Similarly, the ALP-like activity of ZIF-8 was investigated by the cascade reactions of AAP and DTNB. ZIF-8 can catalyze the AAP to generate AA, which will further react with DTNB to generate TNB. A 4 mg sample of ZIF-8, 50 μL of ultra-pure water, 100 μL of 7.3 mM of AAP (prepared in 10.0 mM of phosphate buffer, pH 8.0), and 400 μL of 1.1 mM of DTNB (prepared in ethanol) were mixed in a 1.5 mL centrifuge tube, which was then incubated at 60 °C for 12 min. The absorbance (405 nm) of the supernatant was measured after the mixture had been centrifuged on a mini centrifuge for 3 min. Each sample was measured three times.

### 2.5. Measurement of the K_m_

The Michaelis–Menten constant (*K_m_*), which can be calculated through the Lineweaver–Burk Equation (1) [10], is one of the key parameters of an enzyme kinetic reaction.

(1) $$\frac{1}{V}=\frac{K_{m}}{V_{\max\;}\left[S\right]}+\frac{1}{V_{\max}}$$

where *V* is the initial reaction velocity, which was monitored through the absorbance of TNB (product), and *V_max_* and [*S*] are the maximum reaction velocity and the concentration of substrate (ATCh or AAP), respectively. The experiments were carried out by altering the concentration of ATCh (0.3−1.3 mM) and AAP (0.9−3.6 mM) under optimal conditions.

### 2.6. Procedure for the Determination of Cu^2+^ and AAO

Based on the AChE-like activity of ZIF-8, a simple method for Cu^2+^ detection was developed. In brief, 4 mg of ZIF-8, 50 μL of Cu^2+^ (1.3, 5.2, 20.8, 83.3, 166.7, and 250.0 µM), 150 μL of 11.3 mM of ATCh (prepared in 10.0 mM of phosphate buffer, pH 7.5), and 400 μL of 13.3 mM of DTNB (prepared in ethanol) were mixed in a 1.5 mL centrifuge tube, which was then incubated at 60 °C for 15 min. The absorbance (405 nm) of the supernatant was recorded.

Similarly, based on the ALP-like activity of ZIF-8, a simple method for Cu^2+^ and AAO detection was established. In brief, 4 mg of ZIF-8, 50 μL of Cu^2+^ (4.5, 90.9, 181.8, 272.7, and 454.5 µM) or AAO (2.3, 9.1, 90.9, 181.8, and 454.5 U/L), 100 μL of 7.3 mM of AAP (prepared in 10.0 mM of phosphate buffer, pH 8.0), and 400 μL of 1.1 mM of DTNB (prepared in ethanol) were mixed in a 1.5 mL centrifuge tube, which was then incubated at 60 °C for 12 min. The absorbance (405 nm) of the supernatant was recorded. Each sample was measured three times.

### 2.7. Real Sample Analysis

For the detection of Cu^2+^ in tap water, 4 mg of ZIF-8, 50 μL of Cu^2+^ (with final concentrations of 1.3, 41.7, and 166.7 µM) spiked tap water, 150 μL of 11.3 mM of ATCh (prepared in 10.0 mM of phosphate buffer, pH 7.5), and 400 μL of 13.3 mM of DTNB (prepared in ethanol) were mixed in a 1.5 mL centrifuge tube, which was then incubated at 60 °C for 15 min. The spiked recovery of Cu^2+^ in the tap water was calculated through the linear relationship between the inhibition rate and concentration of Cu^2+^.

Similarly, different concentrations of Cu^2+^ and AAO were spiked in human serum and rabbit plasma and analyzed by the developed method. A 4 mg sample of ZIF-8, 50 μL of Cu^2+^ (with final concentrations of 9.1, 181.8, and 454.5 μM), or AAO (with final concentrations of 9.1, 181.8, and 454.5 U/L) spiked in human serum and rabbit plasma samples, 100 μL of 7.3 mM of AAP (prepared in 10.0 mM of phosphate buffer, pH 8.0), and 400 μL of 1.1 mM of DTNB (prepared in ethanol) were mixed in a 1.5 mL centrifuge tube, which was then incubated at 60 °C for 12 min. The spiked recoveries in human serum and rabbit plasma samples of Cu^2+^ and AAO were calculated through the linear relationship between the inhibition rate and concentrations of Cu^2+^ and AAO.

## 3. Results and Discussion

### 3.1. Characterization of ZIF-8 and Feasibility of the Established Method for the Detection of Cu^2+^ and AAO

**Figure 2:**
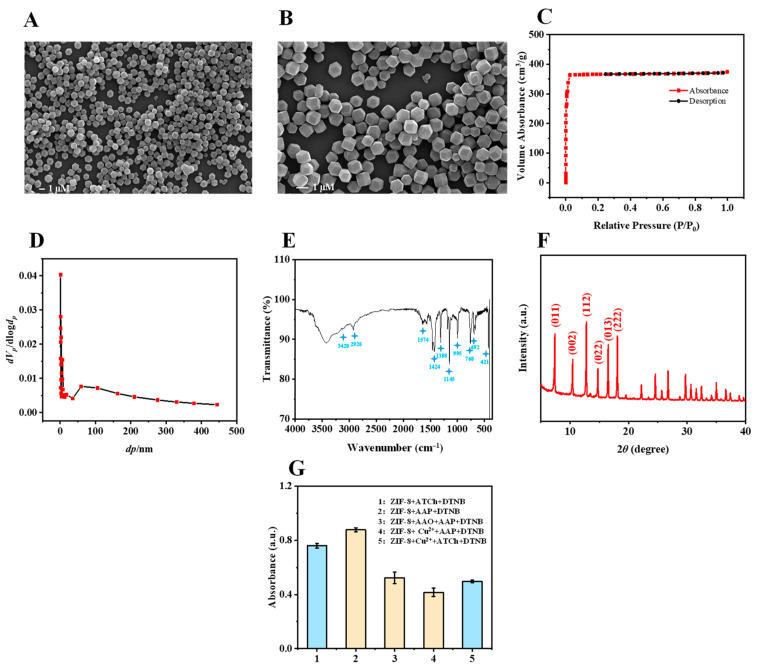
SEM images (**A**,**B**), nitrogen adsorption-desorption isotherm (**C**), pore-size distribution (**D**), FT-IR spectrum (**E**), and X-ray diffraction pattern (**F**) of ZIF-8. The UV absorption (405 nm) of (**G**-1) ZIF-8 + ATCh + DTNB, (**G**-2) ZIF-8 + AAP + DTNB, (**G**-3) ZIF-8 + AAO + AAP +DTNB, (**G**-4) ZIF-8 + Cu^2+^ + AAP + DTNB, (**G**-5) ZIF-8 + Cu^2+^ + ATCh + DTNB. Buffer pH, 7.5 for (**G**) (1 and 5), 8.0 for (**G**) (2, 3, and 4); ZIF-8, 4 mg for G; DTNB, 13.3 mM for (**G**) (1 and 5), 1.1 mM for (**G**) (2, 3, and 4); incubation temperature, 60 °C for (**G**); ATCh, 11.3 mM for (**G**) (1 and 5); AAP, 7.3 mM for (**G**) (2, 3, and 4); reaction time, 12 min for (**G**) (2, 3, and 4), 15 min for (**G**) (1 and 5); centrifugation time, 1 min for (**G**) (1 and 5), 3 min for (**G**) (2, 3, and 4); AAO, 454.5 U/L for (**G**) (3); Cu^2+^, 4.5 µM for (**G**) (4), 20.8 µM for (**G**) (5).

The structure of ZIF-8 was confirmed by SEM (Figure 2A,B), which shows a rhombic dodecahedron of uniform size. The ZIF-8 shows a typical type I isotherm (according to the IUPAC). The Brunauer–Emmett–Teller (BET) surface area of ZIF-8 is 1486.1 m^2^/g with a total pore volume of 0.5779 cm^3^/g and an average pore diameter of 1.6 nm (Figure 2C,D). The FT-IR spectrum of ZIF-8 is displayed in Figure 2E. The absorption peaks at 3420 cm^−1^ and 2926 cm^−1^ are due to the NH and CH stretching vibrations, respectively. The peak at 1574 cm^−1^ is assigned to the CN stretching vibration. The peaks at 1145 and 1308 cm^−1^ belong to the bending signals of the imidazole ring, and the band at 421 cm^−1^ is attributed to the Zn-N stretching vibration [11]. The XRD results show that the characteristic peaks (2*θ* = 011°, 002°, 112°, 022°, and 222°) of the ZIF-8 sodalite topology are consistent with those previously reported [11] (Figure 2F). These results indicate the successful synthesis of ZIF-8.

As shown in Figure 2G, the mixture of ZIF-8, ATCh, and DTNB has an obvious absorption at 405 nm, which is weakened by the addition of Cu^2+^. In addition, the mixture of ZIF-8, AAP, and DTNB also has an absorption at 405 nm after reacting for a certain time, which can be significantly inhibited by the addition of AAO or Cu^2+^. Therefore, it is feasible to detect AAO and Cu^2+^ based on the ALP- and AChE-like activities of ZIF-8.

### 3.2. Optimization of Reaction Conditions

**Figure 3:**
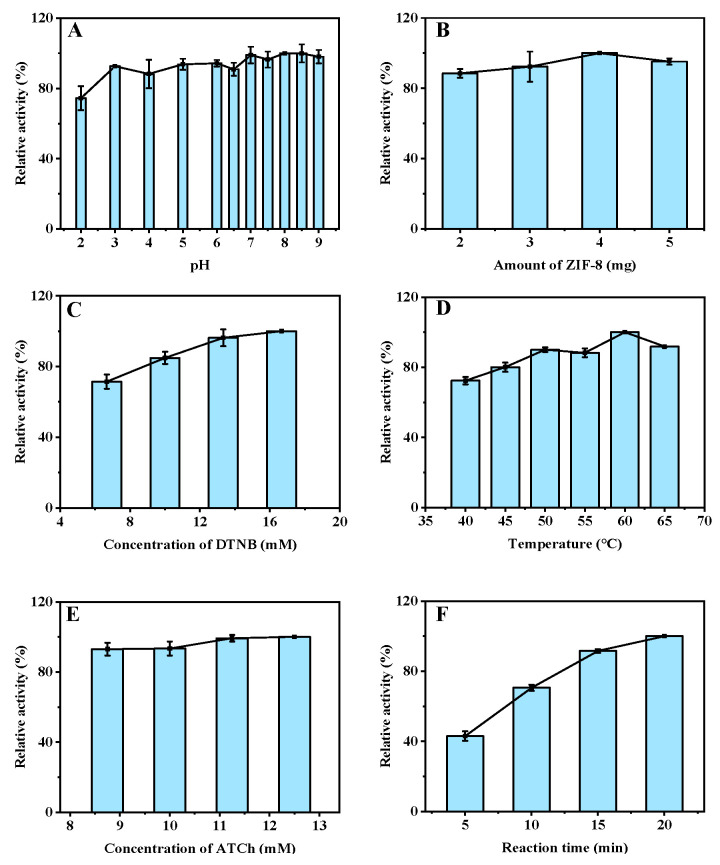
Effects of buffer pH (**A**), amount of ZIF-8 (**B**), concentration of DTNB (**C**), incubation temperature (**D**), concentration of ATCh (**E**), and reaction time (**F**) on the AChE-like activity of ZIF-8 for the detection of Cu^2+^. Buffer pH, 7.5 for (**B**–**F**); ZIF-8, 2 mg for **A**, 4 mg for (**C**–**F**); DTNB, 6.7 mM for (**A**,**B**,**D**–**F**); incubation temperature, 50 °C for (**A**–**C**), 60 °C for (**E**,**F**); ATCh, 5.0 mM for (**A**–**D**), 11.3 mM for and F; reaction time, 5 min for (**A**–**E**); centrifugation time, 1 min for (**A**–**F**).

To ensure a good sensitivity for Cu^2+^ detection based on the AChE-like activity of ZIF-8, experimental parameters of the reaction, such as the buffer pH, amount of ZIF-8, concentrations of DTNB and ATCh, temperature, and reaction time were systematically investigated. The catalytic activity of ZIF-8 keeps steady at a buffer solution pH of 3.0−9.0, indicating its good catalytic activity at a wide pH range. A pH of 7.5, which is close to the physical condition of humans, was selected for the subsequent experiments (Figure 3A) (The maximum point in each curve is set as 100%). Furthermore, the enzymatic activity of ZIF-8 increases with the increase in its amount from 2 to 5 mg and remains steady above 4 mg, which was finally selected for the subsequent experiments (Figure 3B). Moreover, the catalytic activity of ZIF-8 increases with the increase in the concentration of DTNB from 6.7 to 16.7 mM, and 13.3 mM was selected due to the similarity between its result and that of 16.7 mM (Figure 3C). In addition, as the incubation temperature was increased from 40 to 65 °C, the AChE-like activity of ZIF-8 reached the highest at 60 °C, which was selected for the subsequent experiments (Figure 3D). The concentration of ATCh (from 8.8 to 12.5 mM) has a slight impact on the catalytic activity of ZIF-8, and 11.3 mM was chosen for the relatively good activity at this concentration (Figure 3E). Finally, the effect of reaction time (from 5 to 20 min) was investigated, and 15 min was selected for the good activity with a relatively short duration (Figure 3F). Therefore, the optimized reaction conditions for the detection of Cu^2+^ based on the AChE-like activity of ZIF-8 are as follows: The buffer pH is 7.5, the amount of ZIF-8 is 4 mg, the concentrations of DTNB and ATCh are 13.3 mM and 11.3 mM, respectively, the incubation temperature is 60 °C, and the reaction time is 15 min.

**Figure 4:**
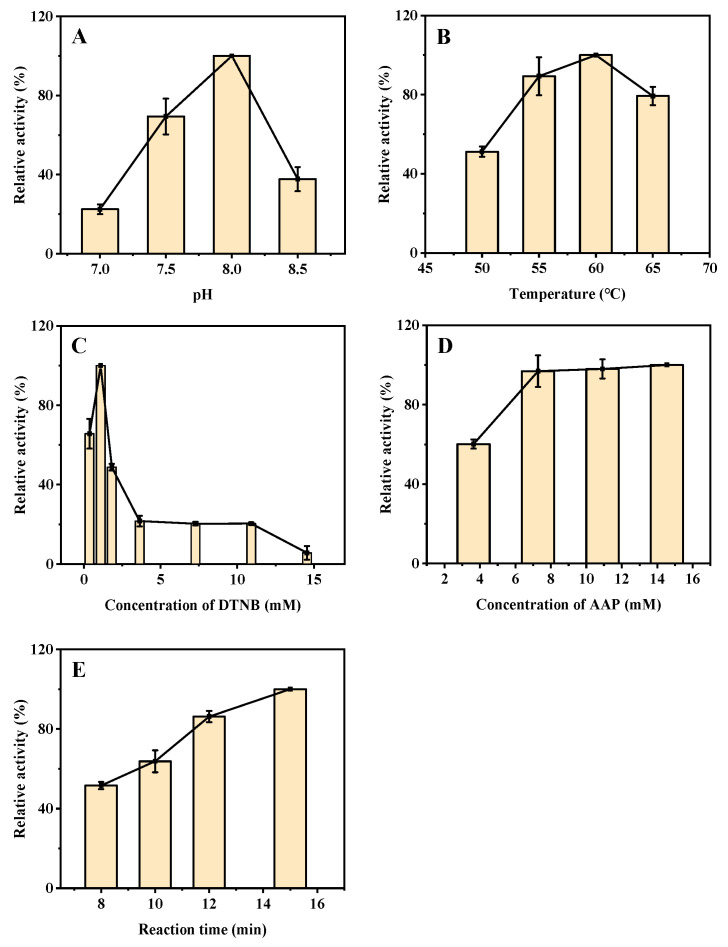
Effects of buffer pH (**A**), incubation temperature (**B**), concentration of DTNB (**C**) and AAP (**D**), and reaction time (**E**) on the ALP-like activity of ZIF-8 for the detection of Cu^2+^ and AAO. Buffer pH, 8.0 for (**B**–**E**); ZIF-8, 4 mg for **A**–**E**; DTNB, 7.3 mM for (**A**,**B**,**D**,**E**); incubation temperature, 60 °C for (**A**,**C**–**E**); AAP, 14.5 mM for **A**–**C**, 7.3 mM for and E; reaction time, 8 min for (**A**–**D**); centrifugation time, 3 min for (**A**–**E**).

Similarly, to ensure a good sensitivity for AAO and Cu^2+^ detection based on the ALP-like activity of ZIF-8, the effect of buffer pH, incubation temperature, the concentrations of DTNB and AAP, and reaction time were thoroughly investigated. The results indicate that the ALP-like activity of ZIF-8 is the highest at a buffer pH of 8.0 (from 7.0 to 8.5), which was selected for subsequent experiments (Figure 4A). Furthermore, the catalytic activity increases as the incubation temperature is increased from 50 °C to 60 °C and decreases above 60 °C (Figure 4B). Therefore, the subsequent tests were performed at an incubation temperature of 60 °C. Moreover, the effect of DTNB concentration on the catalytic activity of ZIF-8 was investigated. There was a phenomenon that high concentrations of DTNB can inhibit the catalytic activity of ZIF-8, probably because too much DTNB will affect the contact between ZIF-8 and the substrate AAP. The catalytic activity of ZIF-8 is the highest at the DTNB concentration of 1.1 mM, which was selected for the subsequent experiments (Figure 4C). Similarly, as shown in Figure 4D, high AAP concentrations (higher than 7.3 mM) had a negative effect on the catalytic activity of ZIF-8, therefore, a relatively low concentration of 7.3 mM was selected. Finally, the effect of reaction time (from 8 to 15 min) on the ZIF-8 catalytic activity was investigated. The catalytic activity increased slowly above 12 min, so 12 min was selected for the follow-up studies (Figure 4E). Therefore, the optimized reaction conditions for the detection of Cu^2+^ and AAO based on the ALP-like activity of ZIF-8 are as follows: The buffer pH is 8.0, the concentrations of DTNB and AAP are 1.1 mM and 7.3 mM, respectively, the incubation temperature is 60 °C, and the reaction time is 12 min.

### 3.3. Kinetics Study of ZIF-8

**Figure 5:**
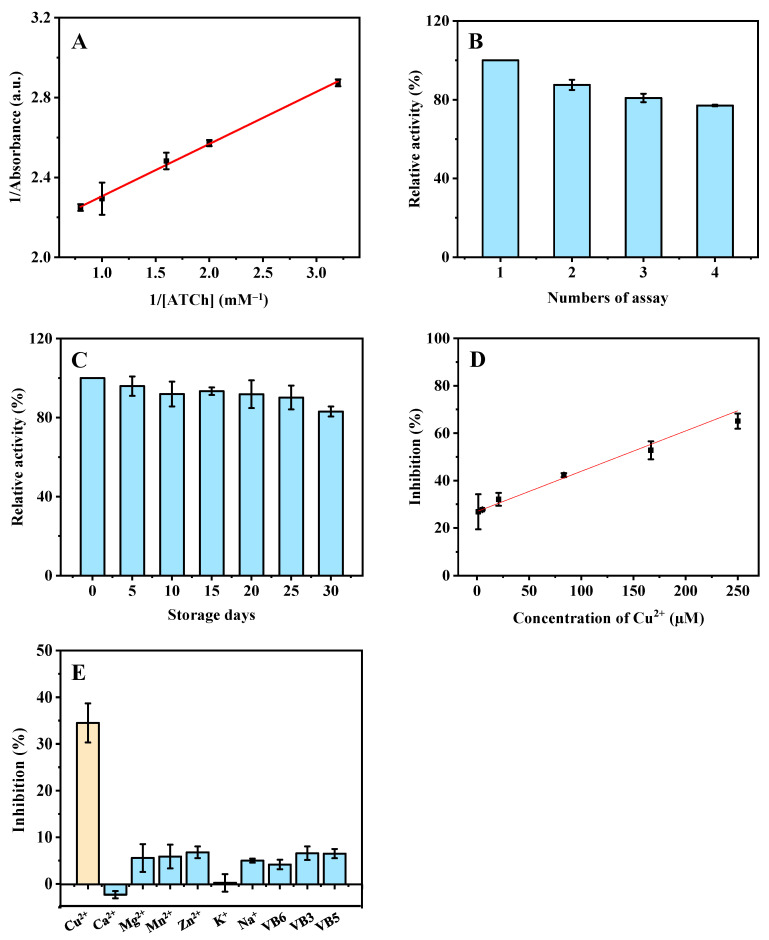
Double reciprocal plot (**A**), the reusability (**B**), storage stability (**C**), linearity (**D**), and the selectivity (**E**) for the detection of Cu^2+^ based on the AChE-like activity of ZIF-8 under the optimized conditions. ZIF-8, 4 mg; buffer pH, 7.5; reaction time, 15 min; centrifugation time, 1 min for (**A**–**E**); ATCh concentrations are from 0.3 to 1.3 mM for (**A**). Cu^2+^ concentrations are from 1.3 to 250.0 µM for (**D**); the concentrations of Cu^2+^ and other substances are 20.8 µM and 83.3 µM for (**E**), respectively.

To investigate the intrinsic AChE-like catalytic activity of ZIF-8, the *K_m_* was determined by measuring the absorbance of the product (TNB) at various substrate (ATCh) concentrations (0.3, 0.5, 0.6, 1.0, and 1.3 mM). According to Equation (1), the acquired linear regression equation of the Lineweaver–Burk plot is *y* = 0.2617 *x* + 2.0442 (R^2^ = 0.9975), where *x* is the reciprocal of ATCh concentration and *y* is the reaction velocity (the absorbance of TNB) (Figure 5A). The *K_m_* (0.1 mM) was calculated via the slope and intercept and differs from that of natural AChE (1.2 mM [12] and 1.0 mM [13]).

**Figure 6:**
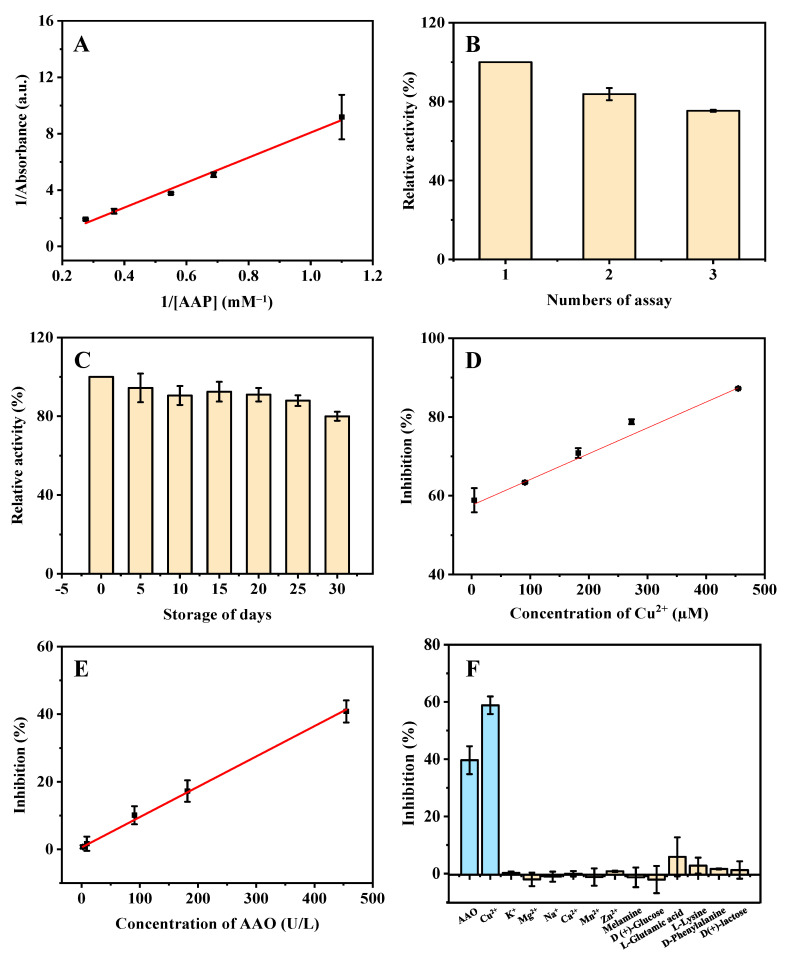
Double reciprocal plot (**A**), the reusability (**B**), storage stability (**C**), linearity of Cu^2+^ (**D**) and AAO (**E**), and the selectivity (**F**) for the detection of Cu^2+^ and AAO based on the ALP-like activity of ZIF-8 under the optimized conditions. ZIF-8, 4 mg; buffer pH, 8.0; reaction time, 12 min; centrifugation time, 3 min for (**A**–**F**); AAP concentrations are from 0.9 to 3.6 µM for (**A**); Cu^2+^ concentrations are from 4.5 to 454.5 µM for (**D**); AAO concentrations are from 2.3 to 454.5 U/L for (**E**). The concentrations of Cu^2+^, AAO, and other substances are 4.5 µM, 454.5 U/L, and 90.9 µM for (**F**), respectively.

In addition, to investigate the intrinsic ALP-like catalytic activity of ZIF-8, the *K_m_* was measured via the absorbance of the product (TNB) at various substrate (AAP) concentrations (0.9, 1.5, 1.8, 2.7, and 3.6 mM). The obtained linear regression equation of the Lineweaver–Burk plot is *y* = 8.8691*x* – 0.7946 (R^2^ = 0.9916), where *x* is the reciprocal of AAP concentration and *y* is the reaction velocity (the absorbance of TNB) (Figure 6A). The determined value of *K_m_* is 11.2 mM, which is similar to that of natural ALP (substrate: disodium phenyl phosphate, 3.8 mM) [14].

### 3.4. Stability of ZIF-8

The AChE-like activity of ZIF-8 has good reusability, retaining about 80.0% relative activity after four repeated cycles (Figure 5B). The ALP-like activity of ZIF-8 also has good reusability, retaining about 80.0% relative activity after three repeated cycles (Figure 6B). The good stability means that ZIF-8 can be reused, which is helpful to reduce experimental costs. Moreover, a batch of ZIF-8 was stored at room temperature for 30 days under dry conditions. The ZIF-8 retained more than 80.0% of its initial enzyme-like activity after 30 days of storage, and the relative standard deviations (RSD) of batch-to-batch (*n* = 3) were 4.0% and 2.7%, respectively (Figure 5C and Figure 6C). These results show that the ZIF-8 has good reusability and storage stability.

### 3.5. Colorimetric Detection of Cu^2+^ and AAO

Finally, the developed methods were used to detect AAO and Cu^2+^ based on the AChE- and ALP-like activities of ZIF-8. A linear dependence was acquired in the range of 1.3−250.0 μM (calibration curve: *y* = 0.1702*x* + 26.9027, R^2^ = 0.9889) (Figure 5D) (AChE-like activity-based) and 4.5−454.5 μM (*y* = 0.06565*x* + 57.5369, R^2^ = 0.9965) (Figure 6D) (ALP-like activity based) for Cu^2+^, respectively. The LODs are calculated to be 0.7 and 2.8 µM, respectively, which are much lower than the drinking water guideline (31.5 μM) of the World Health Organization (WHO) [15], and normal levels of blood copper (15.7−23.6 μM) [16]. Compared with other detection methods (Table 1), this method has a higher sensitivity in aqueous solution and a wider linear range. In addition, the synthesis process of the material ZIF-8 is green, simple, and fast.

Good linear dependence was obtained based on the ALP-like activity of ZIF-8 for AAO detection in the range of 2.3−454.5 U/L (calibration curve: *y* = 0.08971*x* + 0.5772, R^2^ = 0.9982) (Figure 6E). The LOD is calculated to be 1.8 U/L. As compared with other detection methods (Table 2), this method has certain advantages in sensitivity, synthesis process, and experimental cost.

To estimate the selectivity of the methods for Cu^2+^ and AAO detection, the influence of various potential interfering substances was studied. The effects of different inorganic ions (Ca^2+^, Mg^2+^, Mn^2+^, Zn^2+^, K^+^, and Na^+^) and vitamins (VB6, VB3, and VB5) (in tap water) on the detection of Cu^2+^ were investigated. As shown in Figure 5E, these common metal ions and vitamins have no obvious effect on the detection of Cu^2+^, indicating the excellent selectivity for Cu^2+^ detection based on the AChE-like activity of ZIF-8. In addition, the influence of various potential interfering substances (in human serum and rabbit plasma), including inorganic ions (Ca^2+^, Mg^2+^, Mn^2+^, Zn^2+^, K^+^, and Na^+^) and biological small molecules (melamine, D (+)-glucose, L-glutamic acid, L-lysine, D-phenylalanine, D (+)-lactose) were studied. As shown in Figure 6F, the other ions and substances have no significant effect on the ALP-like activity of ZIF-8 for AAO and Cu^2+^ detection, which demonstrates that the developed method based on ALP-like activity of ZIF-8 has good selectivity toward Cu^2+^ and AAO.

### 3.6. Detection of Cu^2+^ and AAO in Real Samples

With the high selectivity and selectivity toward Cu^2+^ and AAO, the developed method was further applied in the detection of Cu^2+^ and AAO in real samples. As shown in Table 3, a standard addition method was adopted by adding Cu^2+^ into tap water to reach final concentrations of 1.3, 41.7, and 166.7 µM, and analysis by the developed (AChE-like activity-based) method. The sample spiked recoveries are from 80.0% to 115.4%, and the RSDs (*n* = 3) are from 0.2% to 2.9%. These results indicate the reliability of the developed method for the determination of Cu^2+^ in tap water based on the AChE-like activity of ZIF-8. Furthermore, a standard addition method was adopted by adding Cu^2+^ and AAO into human serum and rabbit plasma samples to reach final Cu^2+^ concentrations of 9.1, 181.8, and 454.5 µM, and final AAO concentrations of 9.1, 181.8, and 454.5 U/L, and analysis by the developed (ALP-like activity based) method. The sample spiked recoveries for human serum and rabbit plasma samples are in the range of 80.0−119.3% with RSD from 1.4% to 7.0% (Table 4). Therefore, the ALP-like activity of ZIF-8 can be utilized for the quantitative detection of Cu^2+^ and AAO in complex biological samples with good recovery and precision.

## 4. Conclusions

In this study, the ALP-like activity of ZIF-8 is reported for the first time, and based on high AChE- and ALP-like activities of ZIF-8, a novel platform for Cu^2+^ and AAO detection was established. ZIF-8 has good reuse and storage stability. It retains about 80.0% relative activity after four (AChE-like activity) and three (ALP-like activity) repeated cycles, which can realize multiple enzyme analyses and save on experimental costs. The ZIF-8 also retains more than 80.0% of its initial enzyme-like activity after being stored at room temperature for 30 days. The synthesis process of ZIF-8 is green, simple, and fast, which can not only be time-saving but also be harmless to the environment. Moreover, the developed method for detecting Cu^2+^ and AAO based on the enzyme-like activities of ZIF-8 has a low LOD (0.7 and 2.8 µM for Cu^2+^, 1.8 U/L for AAO) and wide linear range (1.3−250.0, 4.5−454.5 µM for Cu^2+^, 2.3−454.5 U/L for AAO). Based on the high sensitivity and selectivity, the developed assay was also successfully applied in the quantitative measurement of Cu^2+^ and AAO in tap water, human serum, and rabbit plasma samples without complex pretreatment. The sample spiked recoveries for human serum and rabbit plasma samples are in the range of 80.0−119.3% with RSD from 1.4% to 7.0%. Therefore, it is believed that the results of the present study may contribute to the development of a new type of multifunctional sensor for multiple analytes, such as metal ion detection and enzyme activity assay.

## Figures and Tables

**Table 1:** Comparisons of the previously reported methods and present work for the detection of Cu^2+^.

Material	Detection Method	Material Synthesis Process	Reaction Time (min)	Linear Range (µM)	LOD (µM)	Ref.
h-CDs	Fluorescence	Heated to 180 °C and kept for 12 h; dried in a vacuum oven	12	0−10	0.2	[17]
Ag/Zn-ZIF-8	Fluorescence	Synthesized at room temperature for 20−24 h; dried overnight in a vacuum oven at 80 °C	30	1−20	6.7	[18]
Gr-SnS	Voltammetry	Synthesized at 180 °C for 16 h; dried in a vacuum oven at 65 °C for 24 h; ultrasonication for 1 h	-	1.5−36	0.02	[19]
ZnO-Co_3_O_4_	Colorimetry	Synthesized at room temperature for 24 h; dried in vacuum at 60 °C for 12 h; heated at 350 °C for 3 h	10	2−100	1.1	[20]
Pt/Co_3_O_4_	Colorimetry	Synthesized at 160 °C for 1 h; dried in a vacuum oven at 50 °C for 12 h; calcined in a muffle furnace at 450 °C for 4 h; synthesized at 90 °C for 20 min under constant stirring	15	10−200	4.1	[21]
Amino-coumarin	Fluorescence	Kept stirring for 19 h at room temperature under a nitrogen atmosphere	-	-	0.2	[22]
DNA-templated AgNCs	Fluorescence	Incubated for 1 h under gentle agitation and room temperature against light exposure; mixed for 30 min in the dark at room temperature	5	2−20	0.1	[23]
Benzothiadiazole	Colorimetry	Stirred at 22 °C for 20 h	-	0−6	0.6	[24]
ZIF-8 (AChE)	Colorimetry	Synthesized at 30 °C for 2h; dried in a vacuum oven at 50 °C for 8 h	15	1.3−250.0	0.7	This work
ZIF-8 (ALP)	Colorimetry		12	4.5−454.5	2.8	

**Table 2:** Comparison of the previously reported methods and present work for the detection of AAO.

Material	Detection Method	Material Synthesis Process	Reaction Time (min)	Linear Range (U/L)	LOD (U/L)	Ref.
CuInZnS QDs	Fluorescence	Synthesized at 180 °C for 5 h	10	0.1−5	0.078	[8]
DNA-Au/Ag NC	Fluorescence and colorimetry	Kept in the dark for 4 h	25	10−200	4.8	[25]
Mn@ZnGe NPS	Fluorescence	Synthesized in a Teflon-lined autoclave at 220 °C for 6 h.	5	1250−2500	728	[26]
Au/Ag NCs	Fluorescence	Heated at 37 °C for 2 h	20	5−80	1.7	[27]
CoCOOH-PLPs	Fluorescence	Stirred for 5 min	71	1−20	0.3	[28]
PB NPs	Colorimetry	Synthesized at 60 °C under vigorous stirring for 0.5 h	70	0.25−14	0.21	[29]
MQDs/CoOOH	Fluorescence	Incubated for 24 h at 200 °C in an oven; sonicated for 10 min; collected by centrifugation at 10,000 rpm for 15 min; sonicated for 24 h	55	2−10	0.8	[30]
Carbon dots	Colorimetry	Performed at 180 °C for 12 h		0.04−8	0.012	[31]
ZIF-8 (ALP)	Colorimetry	Synthesized at 30 °C for 2h; dried in a vacuum oven at 50 °C for 8 h	12	2.3−454.5	1.8	This work

**Table 3:** Determination of Cu^2+^ in tap water based on the AChE-like activity of ZIF-8.

Sample	Added (µM)	Founded (µM)	Recovery (%)	RSD (*n* = 3, %)
1	1.3	1.5	115.4	2.9
2	41.7	40.0	95.9	1.9
3	166.7	133.0	80.0	0.2

**Table 4:** Determination of Cu^2+^ and AAO in human serum and rabbit plasma based on the ALP-like activity of ZIF-8.

Sample	Analyte	Added	Found	Recovery (%)	RSD (*n* = 3, %)
Human serum	Cu^2+^	9.1 µM	9.8 µM	107.7	5.4
		181.8 µM	167.9 µM	92.4	1.6
		454.5 µM	441.3 µM	97.1	2.5
	AAO	9.1 U/L	8.6 U/L	94.5	7.0
		181.8 U/L	216.8 U/L	119.3	3.5
		454.5 U/L	390.0 U/L	85.8	1.4
Rabbit plasma	Cu^2+^	9.1 µM	10.1 µM	111.0	1.5
		181.8 µM	215.6 µM	118.6	2.4
		454.5 µM	487.6 µM	107.3	4.0
	AAO	9.1 U/L	7.8 U/L	85.7	4.8
		181.8 U/L	203.8 U/L	112.1	1.8
		454.5 U/L	363.3 U/L	80.0	1.8

## Data Availability

Not applicable.
